# Pulmonary parenchyma becomes a self‐organized dissipative structure in lung cancers and pulmonary inflammatory diseases

**DOI:** 10.14814/phy2.70738

**Published:** 2026-05-13

**Authors:** Yves Lecarpentier, Aurélien Morini, Bruno Tremblay, Christèle Locher, Xénophon Krokidis, Victor Claes, Olivier Schussler, Christophe Locher

**Affiliations:** ^1^ Centre de Recherche Clinique Grand Hôpital de l'Est Francilien Meaux France; ^2^ Service d'Anatomie et Cytologie Pathologiques Grand Hôpital de l'Est Francilien Jossigny France; ^3^ Service de Chirurgie Thoracique et Vasculaire Grand Hôpital de l'Est Francilien Meaux France; ^4^ Service de Pneumologie Grand Hôpital de l'Est Francilien Meaux France; ^5^ Xénomics Paris France; ^6^ Department of Pharmaceutical Sciences University of Antwerp Wilrijk Belgium; ^7^ Département de Chirurgie Thoracique, Hôpital Cochin, Hôpitaux Universitaires Paris Centre Paris‐Descartes Université, Assistance Publique‐Hôpitaux de Paris Paris France; ^8^ Service d'Hépato‐Gastro‐Entérologie Grand Hôpital de l'Est Francilien Meaux France

**Keywords:** dissipative structures, entropy, lung, mechanics, TGF‐β1, thermodynamics

## Abstract

Far‐from‐equilibrium thermodynamics were studied in patients with either lung cancer or pulmonary inflammatory diseases. Histological and mechanical activities were due to myofibroblasts. A study on isolated lung fragments treated with the Huxley formalism showed that mechanical abnormalities were similar in both groups: maximum velocity, isometric tension, maximum efficiency, force of non‐muscle (NM) myosin crossbridge (CB), time stroke (ts), rate constants for CB detachment, and catalytic constant. Only maximum myosin ATPase activity, myosin content, and CB attachment constant were higher in inflammatory processes than in cancers. Contractile samples were open systems. In each group, β‐catenin, NMIIA and B myosins were present. Thermodynamic force (TFo) and flow (TFl) did not show any significant difference between the two groups of patients. In both groups, TFo varied nonlinearly with TFl. This showed that these two systems behaved far‐from‐equilibrium. Entropy production rate was equal to the TFo × TFl product and did not show any significant statistical difference between the two groups. Excess entropy production was negative in both groups which showed that they were self‐organized. In conclusion, the two groups of patients evolved towards self‐organized dissipative structures driven by TGF‐β1, which created a tissue capable of contractility thanks to myofibroblasts generated by inflammation.

## 1 INTRODUCTION

In pulmonary alveolar septa, normal lung parenchyma contains myofibroblasts (Mayer & Leinwand, 1997; Tomasek et al., 2002), but they do not represent an effective contractile tissue. Gabbiani et al. (Gabbiani et al., 1971) have discovered myofibroblasts which are characterized by the “modification of fibroblasts into cells which are capable of an active spasm in rat wound granulation tissue”. Myofibroblasts appear in response to injury and undergo a fibroblast‐myofibroblast transition (Younesi et al., 2024). They tend to become like a contractile cell in the case of fibrous injury and cancer (Vallee & Lecarpentier, 2019). The conductor of this transition is the Transforming Growth Factor‐β1 (TGF‐β1) (Lakshmi et al., 2017; Wei, Bhattacharyya, et al., 2012). Activation of TGF‐β1 stimulates the canonical WNT/β‐catenin pathway. Among many other examples, fibrotic disorders are characterized by upregulation of TGF‐β1 in lungs (Coker et al., 2001), bronchoalveolar lavage from patients with scleroderma (Ludwicka et al., 1995), type 2 diabetes (Sharma et al., 1997), and glomerular and tubulo‐interstitial diseases (Yamamoto et al., 1996).

Myofibroblasts acquire all the contractile properties observed in striated (cardiac or skeletal) and smooth muscles (Lecarpentier, Tremblay, et al., 2024): (1) they obey Frank‐Starling's law (Starling & Visscher, 1927); (2) they present the hyperbolic tension‐velocity relationship of Hill (Hill, 1938); (3) one observes the three‐dimensional time‐independent tension‐velocity‐length relationship characterizing the level of contractility (Brutsaert & Claes, 1974); (4) relaxation is obtained by inhibition of contractile proteins (Lecarpentier et al., 2013). The mechanical properties of myofibroblasts are due to the presence of non‐muscle myosin (NMIIA and NMIIB or MYH9 and MYH10), which are ultraslow contractile proteins and α‐smooth muscle actin (α‐SMA) (Conti & Adelstein, 2008).

The aim of this study was to determine whether contractile abnormalities were similar in both lung cancers and pulmonary inflammatory pathologies other than cancer. Moreover, we studied the thermodynamic properties of these two kinds of tissues by determining the histological characteristics and the thermodynamic force (TFo) and flow (TFl). The absence of linearity between these two parameters demonstrated that both pulmonary tissues operated far‐from‐equilibrium. The negativity of their excess entropy production (EEP) showed that the lung pathological parenchyma became self‐organized dissipative structures in both cancerous lung tissues and other pulmonary inflammatory diseases (Kondepudi & Prigogine, 2014; Fox‐Rabinovich & Totten, 2006; Glansdorff et al., 1974; Nicolis, 1977; Prigogine et al., 1974). The WΝΤ/β‐catenin pathway appeared to be the common histological inflammatory characteristic in all these pathologies.

Dissipative structures are open systems that evolve to an ordered state as a result of slight fluctuations and appear far‐from‐equilibrium. They are numerous in the field of physics and chemistry. They include the Belousof‐Zhabotinsky reaction (Zhabotinsky, 1991), Bénard‐Taylor convections, turbulating, hurricanes, vortices (Kondepudi & Prigogine, 2014), and fatigue (Naderi, 2020). In biology, there are the Turing structures (Turing, 1990), chirality, enantiomers and racemization (Kondepudi & Prigogine, 2014; Frank, 1953), rhythms (Goldbeter et al., 2012), and allosteric systems (Goldbeter & Lefever, 1972). In the field of medicine, they have been rarely described, such as in cancer (Lefever & Garay, 1977), bipolar disorder (Goldbeter, 2013), medical tribology (Lecarpentier et al., 2022), myocardial hypoxia (Lecarpentier, Schussler, et al., 2024), and in the uterus outside of pregnancy (Lecarpentier et al., 2025).

## 2 MATERIALS AND METHODS

### 2.1 Ethical statement

This study received a favorable advice from the “Comité de Protection des Personnes (CPP) Sud‐Ouest et Outre‐Mer”, n° IDRCB. 2018‐A01134‐51 (dossier 1‐18‐42). A written consent was signed by the patients.

### 2.2 Specimens

Surgical specimens were obtained from 16 patients with pulmonary adenocarcinoma and 19 patients with pulmonary inflammatory diseases without cancers (hamartoma, pulmonary tuberculosis, pulmonary sarcoidosis, nodular lymphoid hyperplasia, aspergilloma, pneumoconiosis, bullous dystrophy, paraganglioma, benign congenital cyst, chronic sero‐fibrinous pleurisy, benign mesothelial hyperplasia, adenomatous hyperplasia, nonspecific chronic fibro‐inflammatory changes, and organizing inflammatory and fibrous pneumonitis).

### 2.3 Histopathology and immunohistochemistry

Tissue specimens were fixed in 10% buffered formalin and embedded in paraffin. Standard 4‐μm‐thick sections were stained with hematoxylin, eosin, and saffron (HES).

Immuno‐histochemistry was performed on Ventana Bench Mark Ultra using the primary polyclonal antibodies MYH9 (NM‐IIA, FineTest, dilution 1:100; catalog no. FNab05479‐20) and MYH10 (NM‐IIB, FineTest, dilution 1:100; catalog no. FNab05471), the mouse monoclonal antibody β‐catenin (β‐catenin, Cell marque, dilution 1:25; catalog no. 224M‐14), and smooth muscle actin (SMA clone 1A4, Zytomed Systems, dilution 1:100). For positive controls, we used stomach specimens (SMA, β‐catenin), placenta specimens (MYH10), and kidney specimens (MYH9). Suppliers and catalog numbers of these commercial blocks are: Mopec™ Control Slides, Normal Human Tissue, Stomach, Catalog No.: 22‐457‐596; Mopec™ Control Slides, Normal Human Tissue, Placenta, Catalog No.: 22‐457‐731; Mopec™ Control Slides, Normal Human Tissue, Kidney, Catalog No.: 22‐457‐654.

### 2.4 Mechanics of lung samples

Lung samples came from adult patients diagnosed with either a lung cancer or pathologies generating pulmonary fibrosis without cancer. During the surgical procedure, a fragment extracted from the pathological lung was cut off and placed in a flask containing a physiological Krebs–Henseleit solution. It was quickly sent to the laboratory of mechanics. A small fragment of lung tissue having approximately the dimension of 5–8 mm in length and about 1 mm^2^ in section was cut and then installed on the electromagnetic transducer of force and length which was previously described (Lecarpentier et al., 1998). The entire system was bathed in the following Krebs–Henseleit solution: 118 NaCl, 1.2 MgSO_4_.7 H_2_O, 4.7 KCl, 1.1 KH_2_PO_4_, 2.5 CaCl_2_.6H_2_O, 24 NaHCO_3_ and 4.5 D‐glucose and maintained at laboratory temperature (*T*). The solution was bubbled with a gas mixture containing 95% O_2_‐5% CO_2_, which allowed maintaining a pH of 7.4. The lung fragment was preloaded with 1mN. Preload was the load that determines the initial length of the preparation. There was a preload at which the performance of the preparation was optimal. The initial length was called Lo.

**FIGURE 1:**
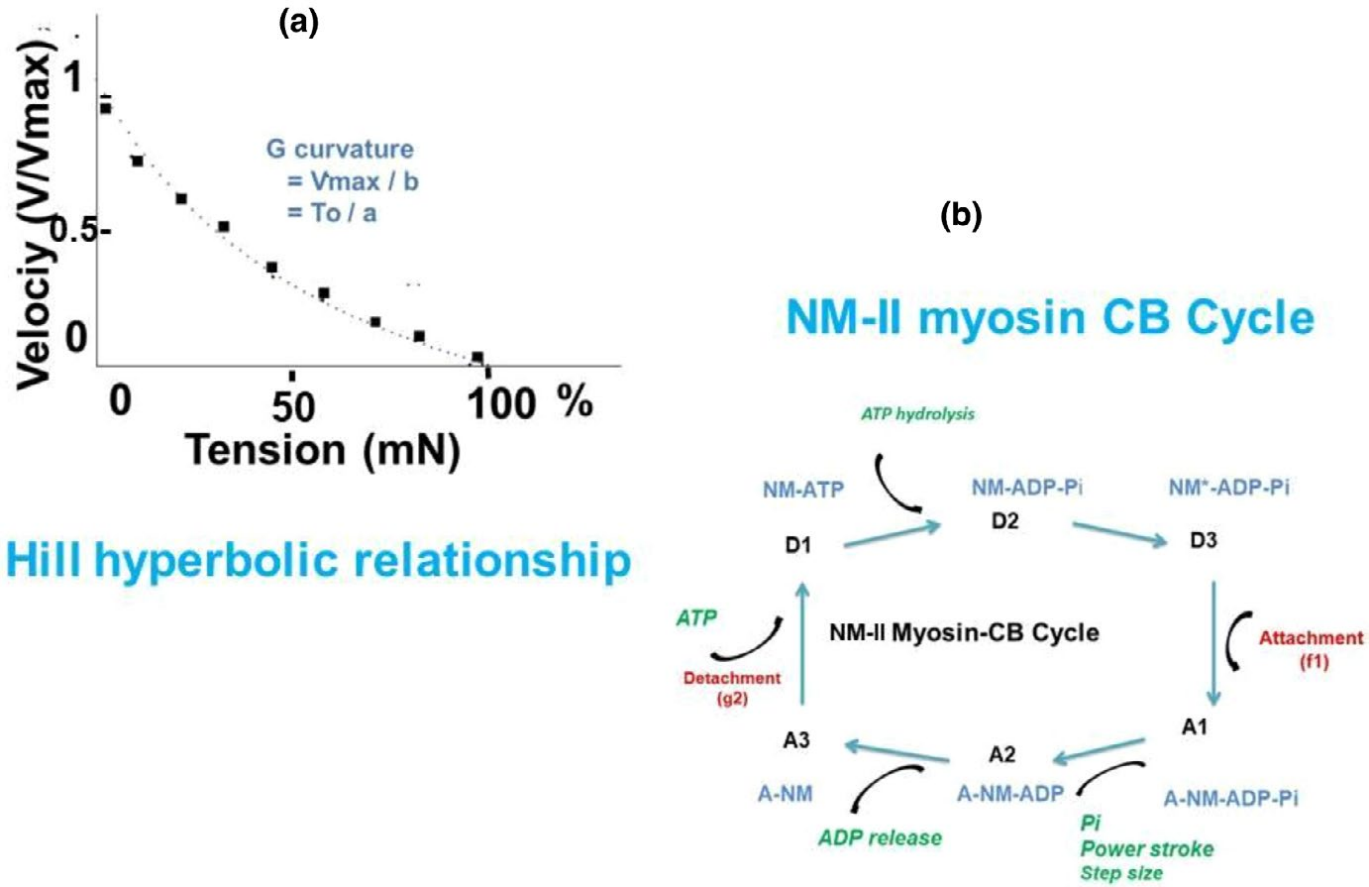
Mechanical and biochemical set‐up. (a) Hill hyperbolic relationship. Maximum shortening velocity was calculated on 9 increasing afterloaded contractions from zero‐load up to isometric tension. (b) NM II‐myosin CB cycle with three detached steps (D1, D2, and D3) and three attached steps (A1, A2, and A3).

The maximum shortening velocity (Vmax in Lo/s) was calculated by the zero‐load clamp technique (Brutsaert & Claes, 1974). The preparation was preloaded to Lo. After 5 ms following electrical stimulation, the preparation was clamped with zero load. In this way, the unloaded shortening velocity was maximal (Vmax). The maximum isometric tension (To in mN/mm^2^) was measured by the peak force normalized by the section of the lung fragment. The Hill hyperbolic relationship (Hill, 1938) was constructed by means of 6 to 9 contractions of increasing isotonic tension (T), from the zero‐load clamp to the isometric tension. This relationship was fitted by a Hill hyperbola: (V + b) (T + a) = b (To + a). The asymptotes of the hyperbola were −a and −b. The G curvature of the hyperbola was Vmax/b = To/a (Figure 1) (Lecarpentier et al., 1998).

### 2.5 A. Huxley formalism

The formalism developed by A. Huxley (Huxley, 1957) is a powerful tool to characterize the molecular properties of contractile proteins, in particular the myosin molecular motor called crossbridge (CB) (Huxley, 1957; Rayment et al., 1993; Spudich, 1994) (Figure 1). The Huxley equations could be applied to the myosin of striated and smooth muscles and to the non‐muscle myosin of myofibroblasts (NMIIA and NMIIB or MYH9 and MYH10). They made it possible to calculate the individual force of a single CB (po in pN), the rate constants for CB attachment (f1 in s^−1^) and CB detachment (g1 and g2 in s^−1^), the catalytic constant (kcat in s^−1^), the myosin content (in nano moles/g of tissue), and the maximum myosin Ca^2+^ ATPase activity which is the product of kcat and myosin content.

The use of Huxley equations required verifying that each biologic sample presented a Hill hyperbolic tension‐velocity relationship. Values for the asymptotes −a and −b of the T‐V relationship were introduced into the Huxley equations. The rate of total energy release (E_Hux_′) and isotonic tension (T = F_Hux_) as a function of velocity (V) were obtained by the following equations:

(1) $$\begin{array}{c} {E_{\mathrm{Hux}}}^{\prime }=\left(N\;e\right)\;\left(h/2\;l\right)\;\left(\text{f1}/\left(\text{f1}+\text{g1}\right)\right)\\ \;\left\{\text{g1}+\text{f1}\;\left(V/\Phi \right)\;\left[\left(1-\exp\;\left(-\Phi /V\right)\right)\right]\right\} \end{array}$$

(2) $$\begin{array}{c} F_{\mathrm{Hux}}=N\;\left(w/l\right)\;\left(\text{f1}/\left(\text{f1}+\text{g1}\right)\right)\;\{1-\left(V/\Phi \right)\\ \;[\left(1-\exp\;\left(-\Phi /V\right)\right)\;\left(1+\left(1/2\right)\right)\;\left(\left(\text{f1}+g1\right)g1/\text{g2}\right)\;\left(V/\Phi \right)]\} \end{array}$$

In these equations, f1 corresponded to the maximum value of the rate constant for NM‐CB attachment; g1 and g2 represented the maximum values of the rate constants for NM‐CB detachment; f1 and g1 corresponded to the tilt of the CB from 0 to h; g2 corresponded to a tilt > h. The NM myosin CB tilt relative to the actin filament varied from 0 to h. This represented the distance of translocation of the actin filament after the NM‐CB swing. The constant l was the distance between two successive actin sites with which a myosin head could bind. A. Huxley conditions imposed that l >> h; h = 10 nm and l = 28.6 nm (Huxley, 1957). Moreover, w was the maximum mechanical work generated by a unitary NM‐CB; e was the free energy to split one ATP and was equal to 10^−19^ J; w/e = 0.75. The standard free energy ΔG°′ATP was −60 kJ/mol; Φ was equal to (f1 + g1) h/2 = b; N was the NM‐CB number per cross‐sectional area.

The parameters po, kcat, f1, g1, and g2 were calculated using the following Equations (Lecarpentier et al., 1998):

(3) $$\mathrm{po}=\left(w/l\right)\times \left[\left(\text{f1}\right)/\left(f1+\text{g1}\right)\right]$$

(4) $$\text{kcat}=\left(h/\text{2l}\right)\times \left[\left(f1\text{g1}\right)/\left(\text{f1}+\text{g1}\right)\right]$$

(5) f1=Gg1

(6) g1=2wb/ehG

(7) g2=2Vmax/h

where kcat was the catalytic constant (in s^−1^) and po was the unitary CB force (in pN). NM myosin content was calculated from the number of cycling myosin CB per mg of tissue and the Avogadro number. The sliding velocity of NM myosin (in μm/s) along actin filaments was vo = h/ts where ts was the time stroke. PA2 was the probability of the time stroke during which the unitary NM‐CB force and the swing of the NM‐CB occurred and was the quotient of ts over tc; tc was the duration of the entire NM‐CB cycle. PA2 was equal to (h/e) po and tc = 1/kcat. The rate of total energy release Eo _Hux_′ under isometric conditions was equal to ab, the product of asymptotes. Thus, after integration, the thermodynamic force was Eo/*T* and its dimension was an energy/*T*. The thermodynamic flow (TFl) was the shortening velocity of the NM‐myosin molecular motor (vo).

### 2.6 General approach of statistical thermodynamics

The first thing to check was that the living pulmonary samples exchanged energy and matter with the outside world, which was the case with human lung samples. We then studied the distance from equilibrium by determining the relationship between the thermodynamic flow and the thermodynamic force. If there was a linear relationship between these two parameters, the system was close to equilibrium. Otherwise, if there was a nonlinear relationship, it was far‐from‐equilibrium.

The product of the thermodynamic force and thermodynamic flow was the entropy production rate (y = EPR):

(8) $$y=\mathrm{EPR}=\delta^{2}S=\left(1/T\right)\times \mathrm{Eo}\times \mathrm{vo}$$

The stability condition was given by the relationship:

(9) $$y^{\prime }=\mathrm{EEP}=\frac{\partial \delta^{2}S}{\mathrm{\partial t}}=\left(1/T\right)\times \mathrm{\delta Eo}\times \mathrm{\delta vo}>0$$

The unstable condition was:

(10) $$y^{\prime }=\mathrm{EEP}=\frac{\partial \delta^{2}S}{\mathrm{\partial t}}=\left(1/T\right)\times \mathrm{\delta Eo}\times \mathrm{\delta vo}<0$$

where $\frac{\partial \delta^{2}S}{\mathrm{\partial t}}$ was the excess entropy production (EEP).

δEo and δvo were the deviations of TFo and TFl from the stationary state, respectively. When the deviation went beyond a critical value, the system reached an instability threshold. A transition to a self‐organized structure was then possible. This was a prerequisite for self‐organization (Fox‐Rabinovich et al., 2018; Gershman et al., 2023; Glansdorff et al., 1974; Nicolis, 1977; Nicolis & Prigogine, 1979).

### 2.7 Determination of a one‐dimensional nonlinear equation with one parameter Ψ

A polynomial regression between y′ and y was then applied to obtain a nonlinear differential equation. A term (G curvature) of this equation was related to y and was introduced in the differential equation. A one‐dimentional nonlinear differential equation with one parameter (Ψ) was then obtained.

### 2.8 Phase diagram and bifurcation diagram

We then studied the phase diagram y′ = f (y, Ψ) and the bifurcation diagram (y = f (Ψ)). On the phase diagram, y′ = f (y, Ψ) showed a non‐hyperbolic fixed point (y*) because λ = f′(y*) was equal to 0. A bifurcation occurred at the non‐hyperbolic fixed point.

### 2.9 Statistics

Box plots are a standardized, graphical way of summarizing the distribution of sets of lung data. They enable the display of five different values–the minimum, first quartile, median, third quartile, and maximum–in a single box shape (Figures 4, 5, 6, 7).

## 3 RESULTS

### 3.1 Histopathology and immunohistochemistry

**FIGURE 2:**
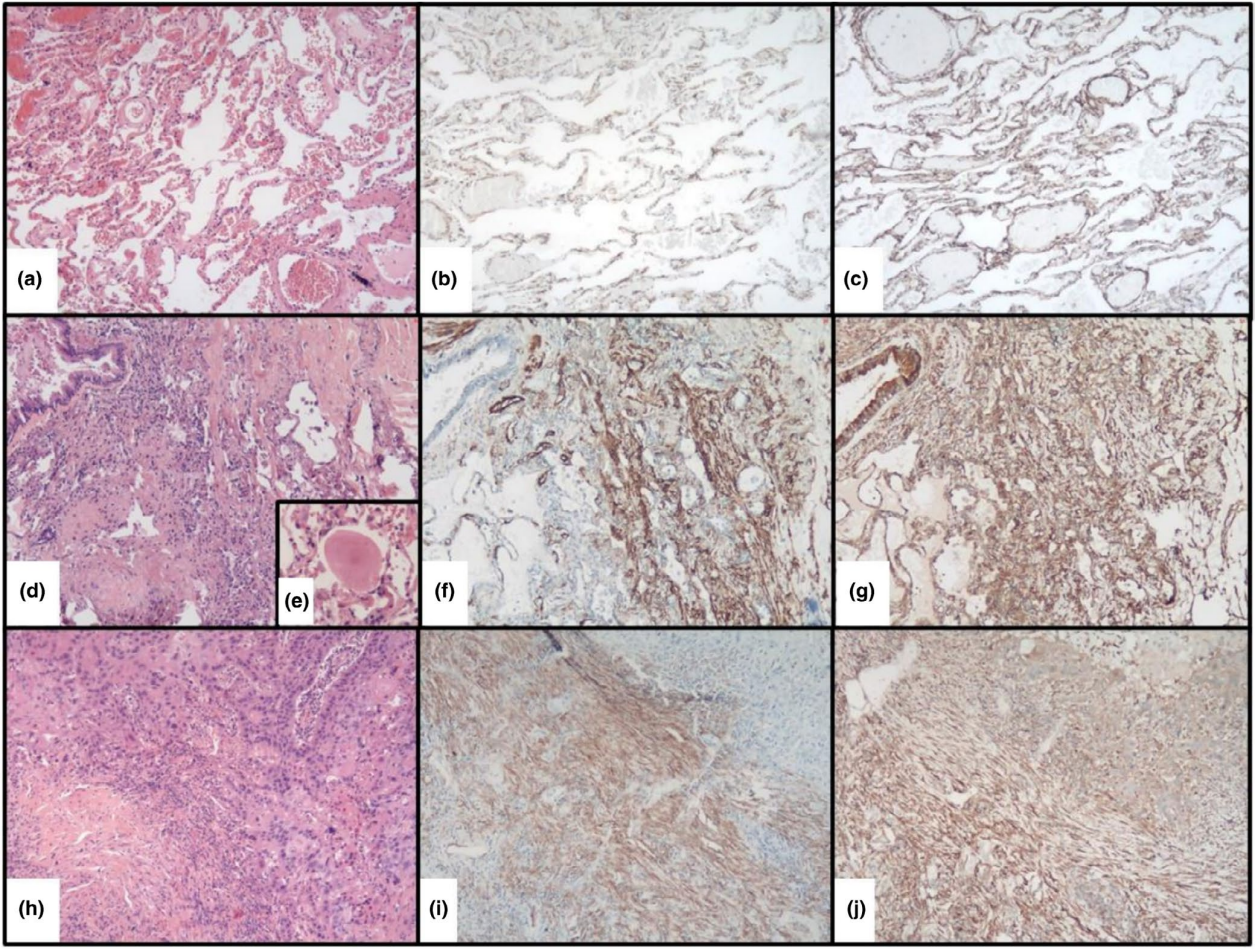
Comparison of immunohistochemical staining of healthy and pathological lungs. Normal lung tissue (a–c) stained with HES (a) with anti‐SMA (b) and anti‐MYH10 (c) immunostaining. Perilesional stroma of an aspergilloma (d–g), stained with HES (d) and showing a cluster of aspergillus filaments in HES (e) with anti‐SMA (f) and anti‐MYH10 (g) immunostaining. Tumor stroma of a bronchopulmonary squamous cell carcinoma (h–j) stained with HES (h) with anti‐SMA (i) and anti‐MYH10 (j) immunostaining. Magnification ×5 for all images.

The interpretation of the slides and the various immunohistochemical markings confirmed the results already known (Lecarpentier, Tremblay, et al., 2024). Healthy lung parenchyma, mainly composed of pulmonary alveoli, showed diffuse and intense expression with β‐catenin, whereas in areas containing stroma, pulmonary myofibroblasts showed much weaker β‐catenin staining and were clearly highlighted by anti‐α‐SMA, MYH9, and MYH10 antibodies, which are naturally expressed in these cells (https://www.proteinatlas.org/) (Figure 2).

**FIGURE 3:**
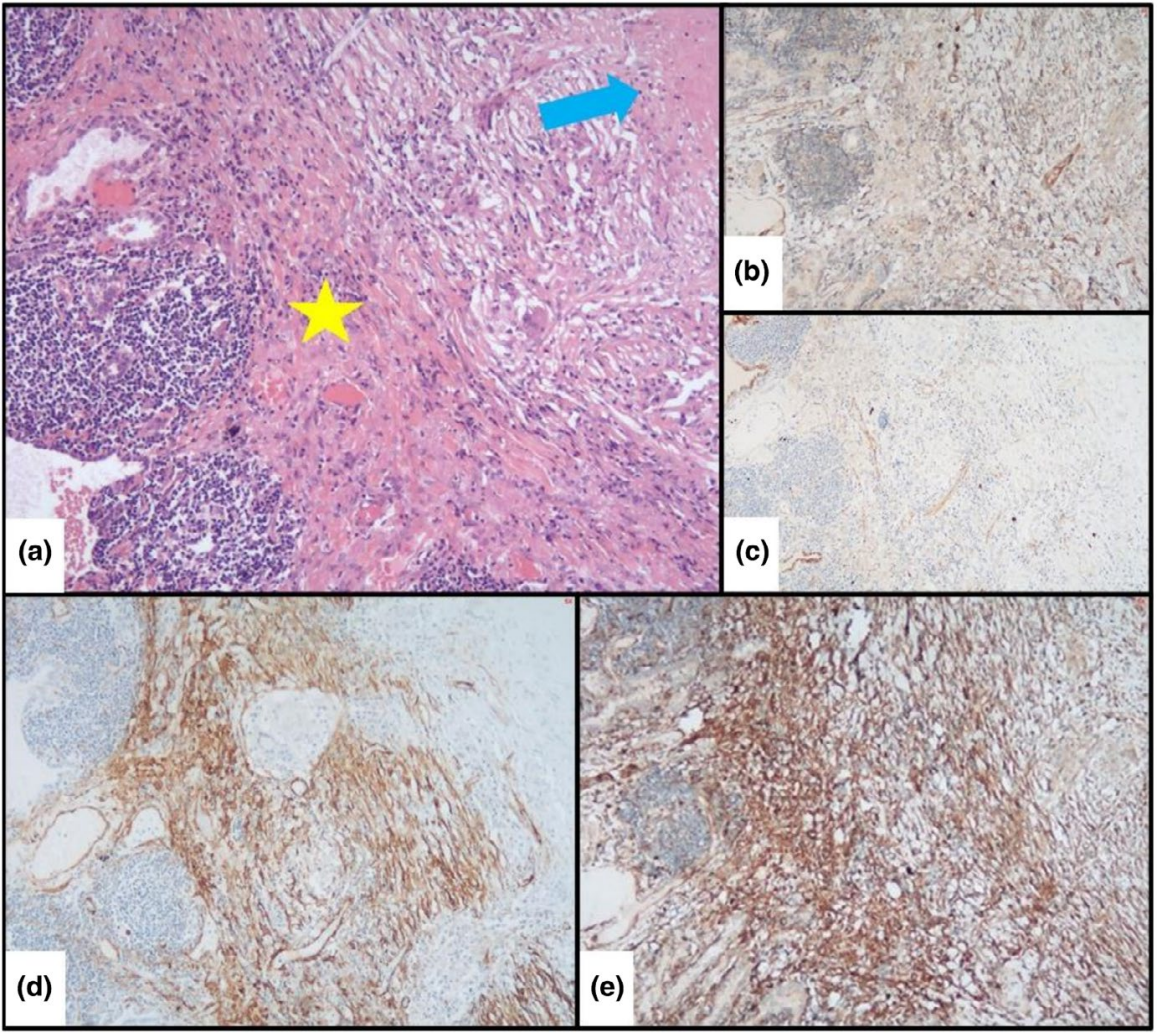
Perilesional stroma of pulmonary tuberculosis stained with HES and with various immunohistochemical markers. Perilesional stroma (yellow star) of pulmonary tuberculosis (blue arrow pointing to the inflammatory infiltrate of tuberculosis and caseous necrosis) stained with HES (a) with anti‐MYHA (b), anti‐β‐catenin (c), anti‐SMA (d), and anti‐MYH10 (e) immunostaining. Magnification ×5 for all images.

As previously described (Lecarpentier, Tremblay, et al., 2024), in tumor cells, membranous staining of β‐catenin appeared, and the pulmonary peritumoral stroma in the two cases tested here (adenocarcinoma and squamous cell carcinoma) shows increased staining with anti‐α‐SMA, MYH9, and MYH10 antibodies due to a much larger population of myofibroblasts within the stroma (Figures 2 and 3). We obtained identical staining patterns with noncancerous pathologies (pulmonary tuberculosis, pulmonary sarcoidosis, nodular lymphoid hyperplasia, aspergilloma, and pneumoconiosis), which was even more significant given that they had a localized, single, even “pseudotumoral” presentation, with weaker staining in more diffuse and interstitial inflammatory pathologies (Table 1).

**TABLE 1:** Expression of α−SMA, β‐catenin, MYH9, and MYH10 in the stroma of tumoral and nontumoral pulmonary lesions.

Specimen (stroma)	α−SMA	β‐Catenin	MYH9	MYH10
Sarcoïdosis	+	+/−	+	++
Nodular lymphoid hyperplasia	+	+/−	+	+++
Aspergilloma	++	+/−	+	++
Pneumoconiosis	+	+/−	+	++
Tuberculosis	++	+/−	+	++
Adenocarcinoma	++	+/−	+	++
Squamous cell carcinoma	++	+/−	+	++

*Note*: Expression of α‐SMA, β‐catenin, MYH9 and 10 in tumoral and non‐tumoral pulmonary diseases.

### 3.2 Mechanics of lung parenchyma and NM myosin crossbridges (CB)

**FIGURE 4:**
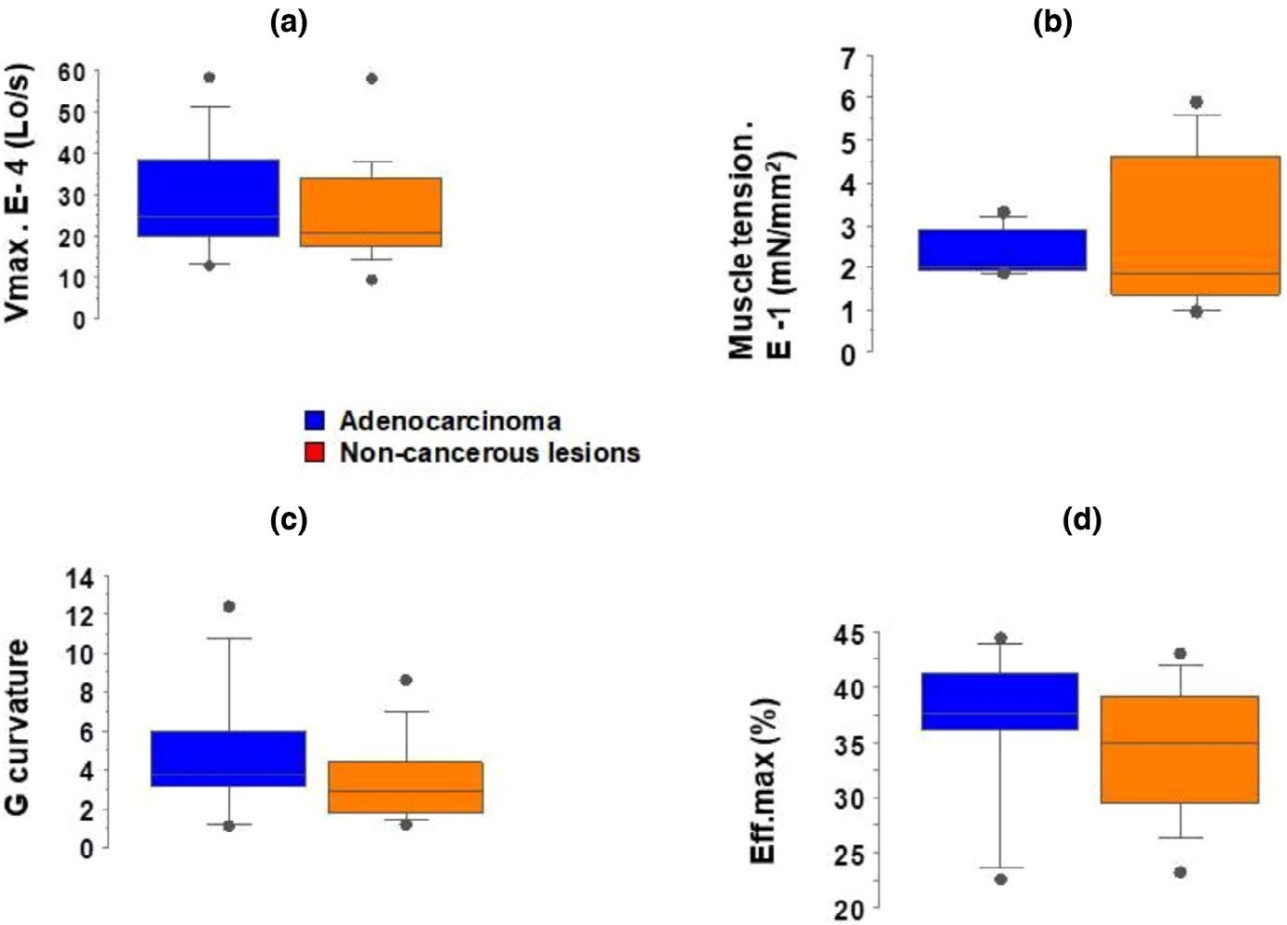
Mechanical parameters of lung samples. Box plot; (a) Maximum unloading shortening velocity (Lo/s); (b) total muscle tension (mN/mm^2^); (c) G curvature of the hyperbolic T‐V relationship (without dimension); (d) Maximum efficiency (Effmax in percentage).

The two groups of lung tissues, namely cancers and noncancerous inflammatory tissues, did not show statistically significant differences in Vmax (Figure 4a), tension (Figure 4b), G curvature of the hyperbolic T‐V relationship (Figure 4c), and efficiency (Figure 4d).

**FIGURE 5:**
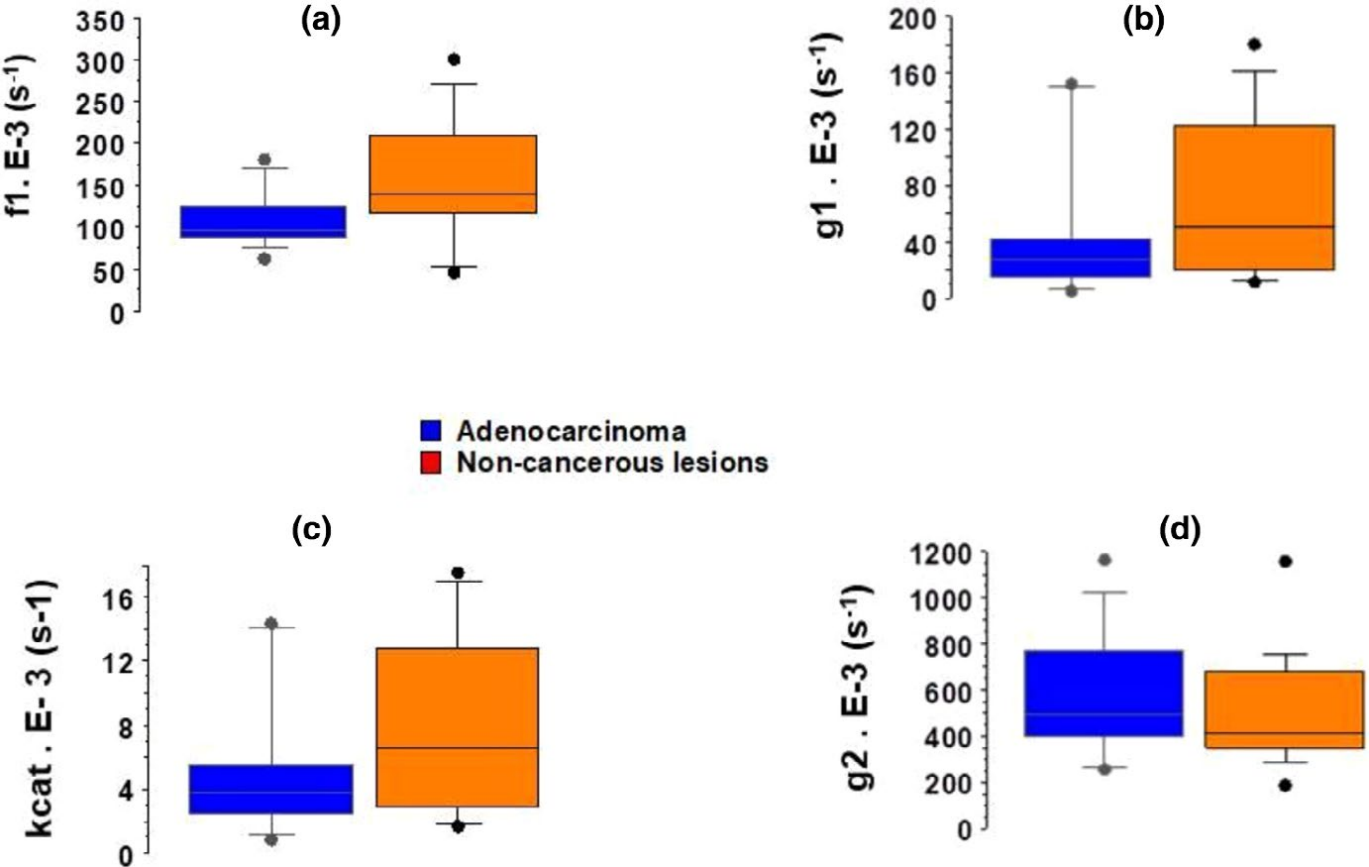
Ultrastructural parameters of NM crossbridges. Box plot: (a) Rate constant for CB attachment (f1 in s^−1^); (b) Rate constant for CB detachment (g1 in s^−1^); (c) NM myosin catalytic constant (kcat in s^−1^); (d) Rate constant for CB detachment (g2 in s^−1^).

**FIGURE 6:**
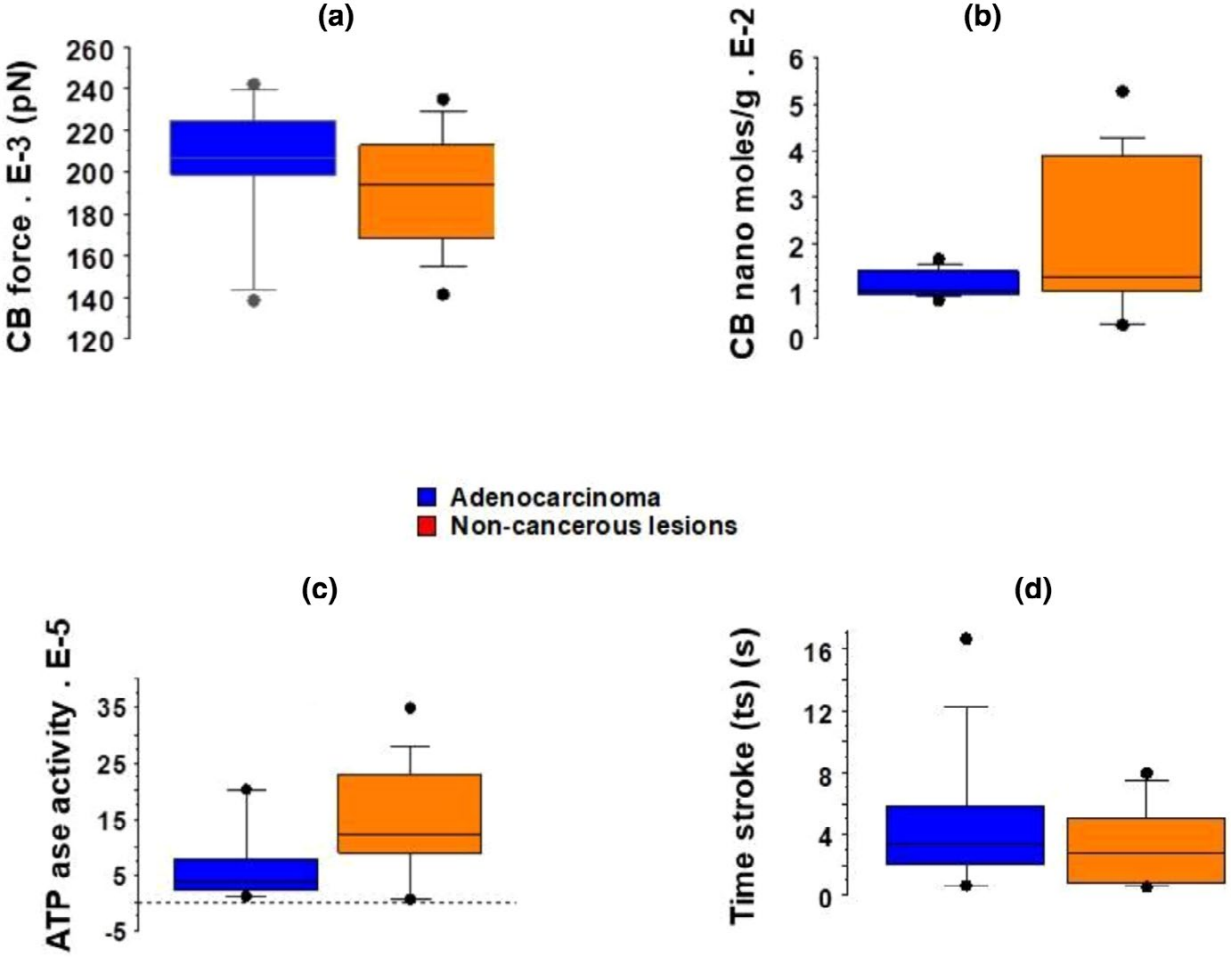
Ultrastructural parameters of NM myosin. Box plot; (a) NM unitary CB force (pN); (b) NM‐myosin content (nano moles/g of tissue); (c) Maximum NM myosin Ca^2+^ ATPase activity (nano moles/g of tissue/s); (d) Time stroke (ts in s).

The following parameters concerning NM myosin crossbridges did not show statistically significant differences: Rate constants for CB detachment g1 (Figure 5b) and g2 (Figure 5d), catalytic constant kcat (Figure 5c), unitary CB force (Figure 6a), and time stroke (Figure 6d). However, the rate constant for CB attachment f1 (Figure 5a), the maximum Ca^2+^ NM myosin ATPase activity (Figure 6c), and the NM myosin content (Figure 6b) were significantly greater in noncancerous inflammatory lung tissues than in purely lung cancers.

### 3.3 Far‐from‐equilibrium thermodynamics

**FIGURE 7:**
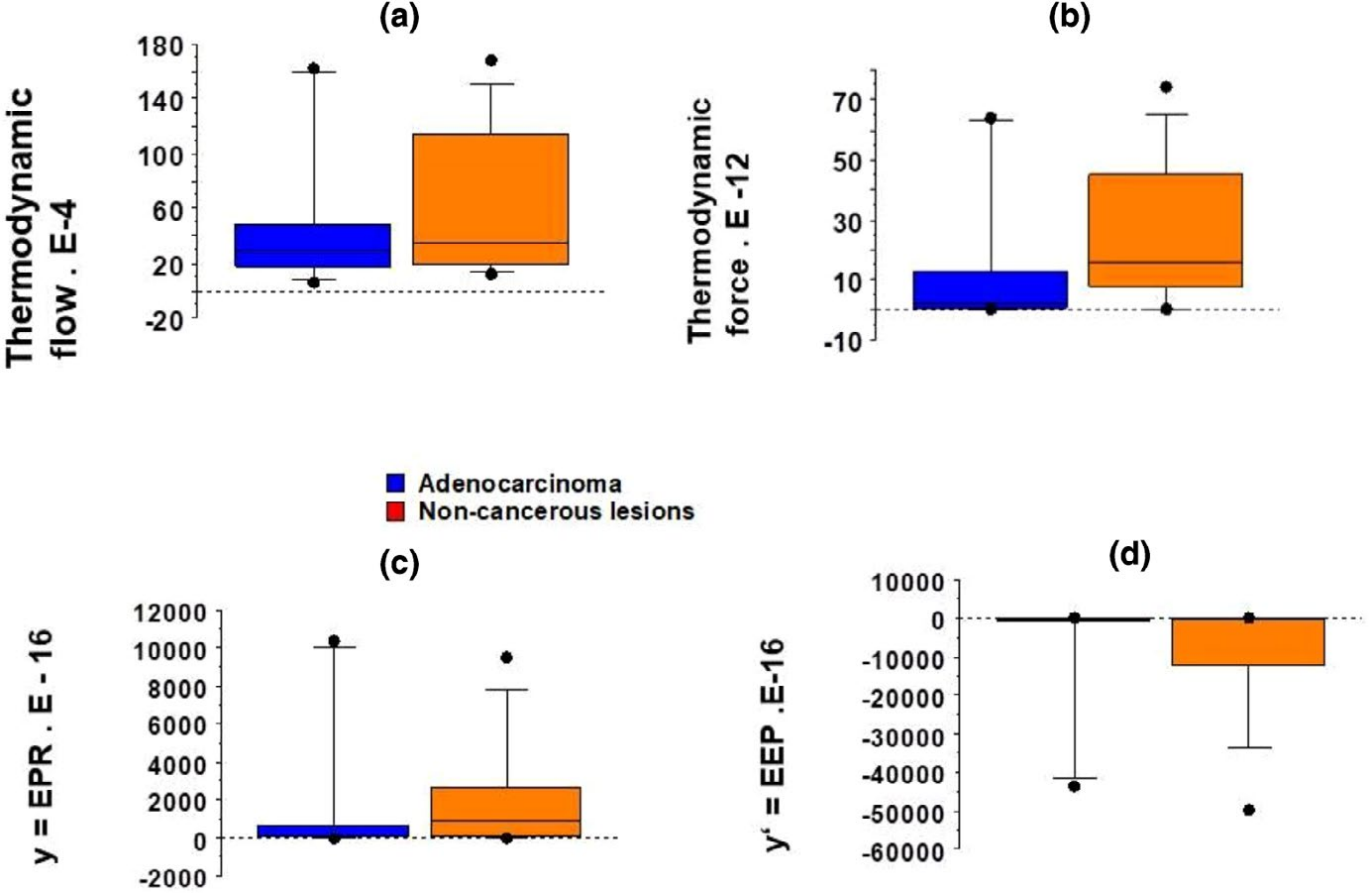
Thermodynamic parameters of lung samples. Box plot; (a) Thermodynamic flow (TFl in s^−1^); (b) Thermodynamic force (TFo = Eo/T, in energy unit/T; in Lo.mN/mm^2^/T); (c) Entropy production rate (y = EPR = TFo × TFl, in Lo. mN/mm^2^/T . s^−1^); (d) Excess entropy production (y′ = EEP = dEPR/dt, in Lo.mN/mm^2^/T.s^−2^).

**FIGURE 8:**
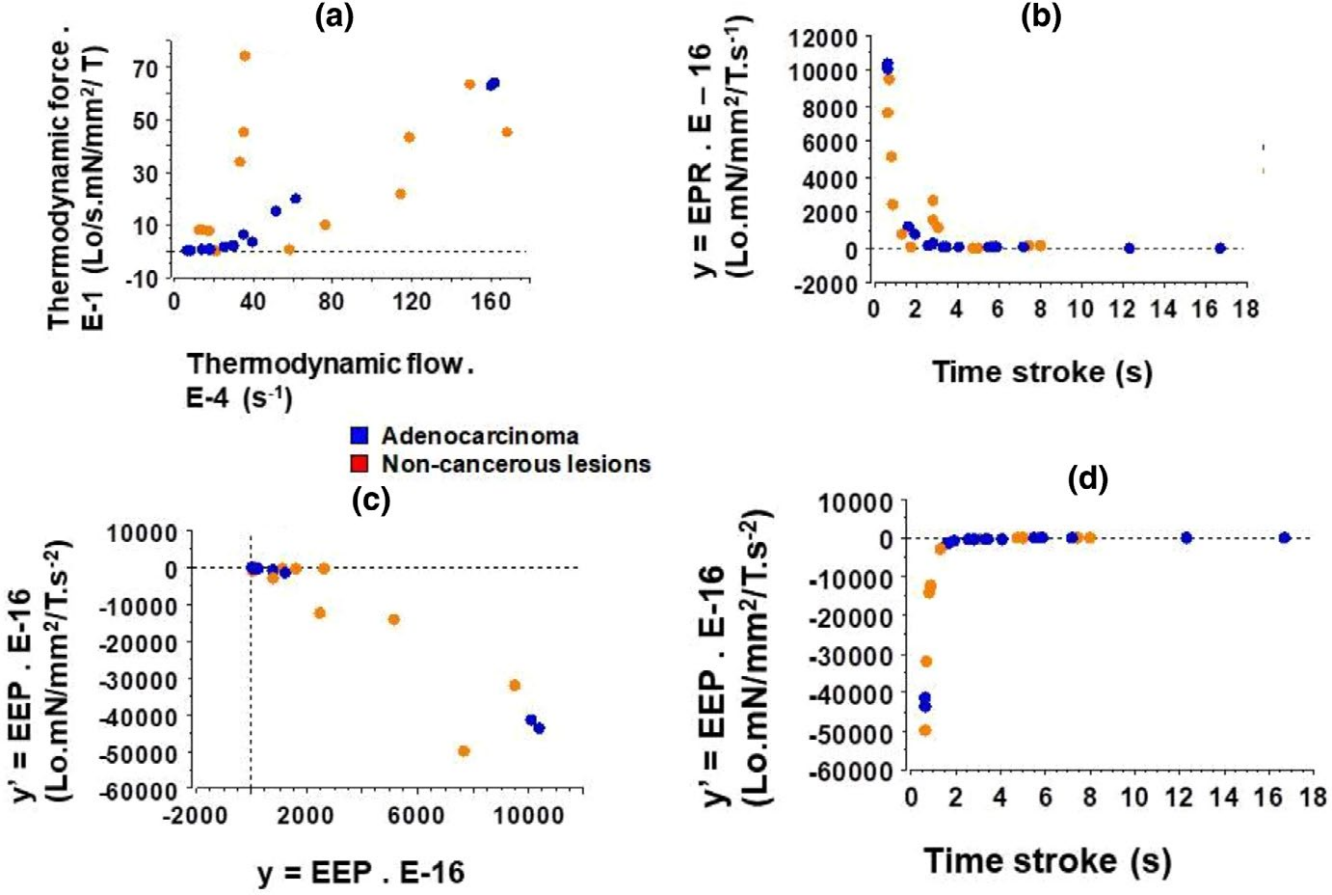
Thermodynamics of lung samples. (a) Thermodynamic force versus thermodynamic flow relationship; (b) Entropy production rate (EPR = TFo × TFl) versus ts relationship; (c) Phase diagram: Excess entropy production (EEP = dEPR/dt) versus entropy production rate relationship; (d) Excess entropy production versus ts relationship.

Thermodynamic force (TFo) was slightly but non significantly greater in noncancerous inflammatory lung tissues than in purely lung cancerous tissues, and TFl did not show statistically significant differences between the two groups (Figure 7a,b). Importantly, there was no linear relationship between thermodynamic force and thermodynamic flow (Figure 8a). This means that both lung systems were in the far‐from‐equilibrium domain. Entropy production rate (y = EPR) (Figure 7c) and excess entropy production (y′ = EEP) (Figure 7d) did not show any statistically significant differences between the two groups. Importantly, EEP was negative in both groups, meaning that both lung tissue groups were in the self‐organized domain.

Figure 8a shows that there was no linear relationship between the thermodynamic force and the thermodynamic flow. The two groups behaved far‐from‐equilibrium.

y = EPR (Figure 8b) and y′ = EEP (Figure 8d) are represented as a function of time stroke.

Excess entropy production (EEP) was negative in both groups which showed that they were self‐organized (Figure 8c).

### 3.4 Determination of a one‐dimensional nonlinear differential equation

**FIGURE 9:**
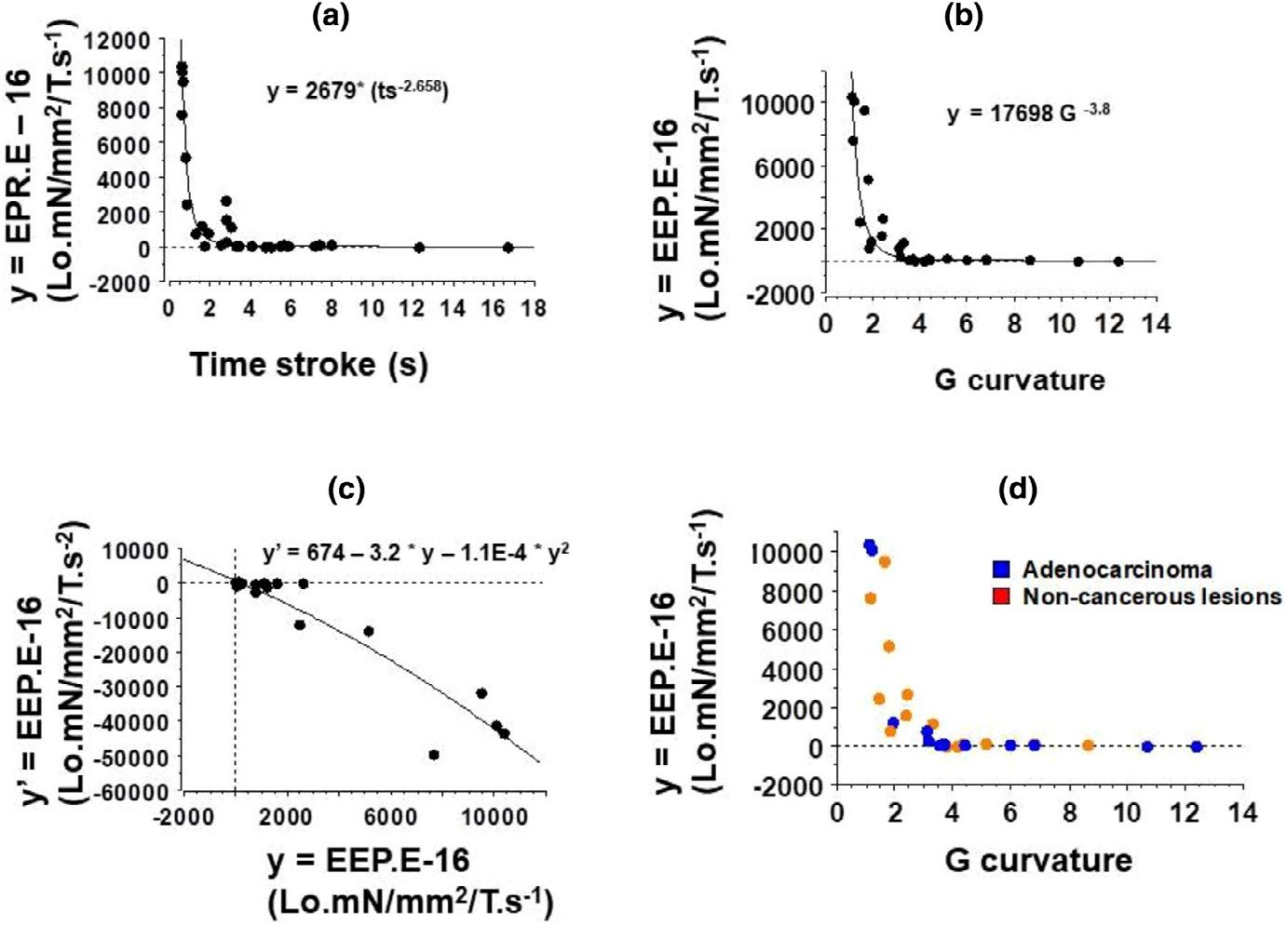
Thermodynamic parameters. (a) Y = EPR versus ts fit; y = 2679 ts^−2.66^; (b) Y versus G curvature fit; y = 17698 G^−3.8^; (c) Phase diagram: y′ versus y fit; y′ = 674−3.2 y−1.1 E‐4 y^2^; (d) Bifurcation diagram (y = f(G)). The bifurcation occurred for approximately G # 3 where y drastically increased as G decreased.

y′ was expressed as a function of y according to the following equation (Figure 9c):

$$y^{\prime }=674-3.2\;y-1.1\;E-4\;y^{2}$$

Moreover, y is a nonlinear power function of G (Figure 9b):

$$y=f\;\left(G\right)=17698\;G^{-3.8}$$

Thus, y′ = 674 − (3.2 × 17698) G^−3.8^ − 1.1 E‐4 y^2^

$$y^{\prime }=-1.1\;E-4\;y^{2}-57\;\text{E3}\;G^{-3.8}+674$$

We obtained a one‐dimensional nonlinear equation with one parameter Ψ = f (G).

### 3.5 Phase diagram and bifurcation diagram

On the phase diagram, y′ = f(y) was always negative, indicating the self‐organizing nature of the system (Figures 8c and 9c). On the bifurcation diagram y = f (Ψ) (Figure 9d), there was a sudden change of direction attesting to the bifurcation for a G value approximating 3.

### 3.6 Stability analysis

The stability analysis was best determined on a case‐by‐case basis, using a graphical method (Figure 8c). The system had a fixed point y* with f′(y*) = 0. The slope of the function y′ = f(y, Ψ) at the fixed point y determined the stability of the system. Thus, y* was a non‐hyperbolic fixed point. Figure 8c showed that the system was unstable and this arose in the context of bifurcation.

## 4 DISCUSSION

The basic histological and mechanical properties of myofibroblasts were not fundamentally different between cancerous lung tissues and inflammatory pathologies of numerous pulmonary etiologies. The canonical WNT/β‐catenin signaling was stimulated and the contractile function operated by means of non‐muscle myosin (NMIIA and NMIIB or MYH9 and MYH10) and α‐SMA.

Table 2 shows the mechanical properties of muscles and non‐muscle structures which were studied under the same conditions thus allowing comparisons. Dissipative structures are shown in blue, and non‐dissipative structures in green and are characterized by their negative excess entropy production. We see that, in addition to inflammatory and cancerous lung pathologies, dissipative structures have been highlighted on muscle structures, on the heart under prolonged anoxia and the uterus outside of pregnancy.

**TABLE 2:** Contractile parameters of NMII and MII myosins.

	Lung inflammation	Lung cancer	Non‐pregnant uterus (Lecarpentier et al., 2025)	Pregnant uterus (Lecarpentier et al., 2025)	Normoxic heart (Lecarpentier, Schussler, et al., 2024)	Hypoxic heart (Lecarpentier, Schussler, et al., 2024)
Vmax	0.003	0.003	0.013	0.034	3.8	2.0
Tension	0.3	0.2	24.5	28.3	51	20
CB force	1.9	2.0	1.2	1.8	1.6	1.2
kcat	0.008	0.004	0.261	0.123	20.6	27.6
G	3.5	4.5	0.9	2.1	1.6	0.9
f1	0.16	0.09	2.63	2.25	314	306
g1	0.07	0.04	3.96	1.04	199	358
g2	0.50	0.52	2.64	6.89	753	398
vo	6.2 E‐3	4.9 E‐3	0.39	0.11	5.1	8.1
MC/T	25.9 E‐12	12.6 E‐12	8.7 E‐3	7.3 E‐3	0.043	0.025
EPR	2236 E‐16	1522 E‐16	3.50	0.47	240 E‐3	200 E‐3
EEP	−7989 E‐16	−5530 E‐16	−0.08	1.82	0.003	−0.002

*Note*: Parameters of contraction and myosin crossbridge characteristics. Vmax: Lmax/s; Tension: mN/mm^2^; kcat: s^−1^; CB force: pN; max.Eff(%): G curvature; f1: rate constant for attachment in s^−1^; g1: rate constant for detachment in s^−1^; g2: rate constant for detachment in s^−1^. Thermodynamic force and flow, entropy production rate (EPR) and excess entropy production (EEP) were also represented.

### 4.1 Myofibroblasts and TGF‐β1 activity

In granulation tissues, modified fibroblasts or myofibroblasts have been reported first by Gabbiani (Gabbiani et al., 1971) and their role in the mechanism of wound healing has been demonstrated. An important ultrastructural characterization of contractile myofibroblasts is the presence of actin filament bundles containing α‐SMA, peripheral focal adhesions, and gap junctions. Myofibroblasts have been described in numerous fibrotic diseases such as idiopathic pulmonary fibrosis, glomerulosclerosis, liver cirrhosis, systemic sclerosis, stromal reaction to epithelial cancer, retinal reaction, and heart failure (Hinz et al., 2012). Myofibroblasts are observed in numerous pathophysiological tissues, including cancer (breast carcinoma, pulmonary cancer, epithelial cells of cancerous mammary glands) and in fibrotic processes (Dupuytren nodules and hypertrophic scars) (Chiavegato et al., 1995; Lecarpentier, Tremblay, et al., 2024). When the canonical WNT pathway is upregulated, as in cancers, the increase in β‐catenin leads to activation of the expression of numerous genes, in particular CYCLIN D1 and cMYC (Lecarpentier et al., 2019). By treating the epithelial cell line with TGF‐β1, this induces a positive expression of α‐SMA and myosin light chain kinase, thus creating a contractile property of the tissue (Islam et al., 2018).

Myofibroblast differentiation is controlled by TGF‐β1, which activates the canonical WNT/β‐catenin pathway (Wei, Bhattacharyya, et al., 2012). TGF‐β1 increases α‐smooth muscle actin (α‐SMA) synthesis, leading to myofibroblast differentiation. Incorporation of α‐SMA in stress fibers stimulates the contractile properties of non‐muscle myofibroblasts (Hinz et al., 2001). The molecular motors are type II non‐muscle myosins (NMIIA and B or MYH9 and MYH10) in non‐muscle contractile cells. The main characteristic of NMIIA and B is their extreme slowness.

Myofibroblasts are key effectors of fibrosis through excessive depositions of collagen and altered extracellular matrix. Fibrosis is evident in numerous lung diseases such as cystic lung disease, granulomatous lung disease, asthma, scleroderma, chronic obstructive pulmonary disease, and sarcoidosis (Wilson & Wynn, 2009). Responsibility of TGF‐β1 activity seems major because inhibiting its activity by the SMAD‐mediated pathway (Flanders, 2004) significantly reduces fibrosis in kidney (Border et al., 1992; Inazaki et al., 2004; Sato et al., 2003), dermis (Flanders et al., 2002; Lakos et al., 2004), eyes (Stramer et al., 2005), and lung (Bonniaud et al., 2004; Zhao et al., 2002). The canonical WNT/β−catenin pathway is hyperactivated in systemic sclerosis and induces Smad‐dependent fibrosis in mesenchymal cells (Wei, Fang, et al., 2012).

### 4.2 Dissipative structures

Dissipative structures are non‐equilibrium open systems, appearing far‐from‐equilibrium and exchanging energy and matter with the external environment (Prigogine, 1986; Prigogine et al., 1969). In these structures, there is no linearity between the thermodynamic flow and the thermodynamic force, which accounts for their far‐from‐equilibrium character. Moreover, excess entropy production is negative, which accounts for their self‐organized character (Kondepudi & Prigogine, 2014; Fox‐Rabinovich et al., 2018; Gershman et al., 2023; Glansdorff et al., 1974; Naderi, 2020; Nicolis, 1977; Prigogine et al., 1974).

The WNT/β‐catenin pathway with the non‐muscular contractile proteins (NMIIA and B or MYH9 and MYH10) and α‐SMA conferred a particular arrangement specific to pulmonary inflammatory and cancerous pathologies, leading to the synthesis of self‐organized dissipative structures. It would be quite likely that this also holds true in other organs such as kidneys, skin, heart, etc., so that any inflammatory syndrome would therefore lead to a dissipative structure with negative EEP.

## 5 CONCLUSION

Lung cancers and non‐cancerous pulmonary inflammatory diseases presented histological and mechanical properties due to the presence of myofibroblasts thank to their non‐muscle myosins. The TGF‐β1 and the WNT/β‐catenin signaling were the common inflammatory pathway and the common trajectory taken by all lung cancerous and pulmonary inflammatory etiologies without cancer. This pathway determined a far‐from‐equilibrium thermodynamic behavior. Self‐organized dissipative structures appeared in all inflammatory pulmonary pathologies studied. The two groups of lung cancers and noncancerous inflammatory pulmonary diseases evolved towards self‐organized structures in which TGF‐β1 created a far‐from‐equilibrium tissue capable of contractility thanks to myofibroblasts. Lung inflammatory diseases led to dissipative structures.

## AUTHOR CONTRIBUTIONS

Conceptualization, Y. L.; data curation, Y. L., O. S., D.F., AM, Christèle. L, X. K. and V.C.; formal analysis, Y. L., B. T. and Chrystèle. L; funding acquisition, Y. L., AM and Christophe. L.; methodology, Y. L., O. S., V. C., AM and D. F.; project administration, Christèle. L, Christophe. L.; resources, Y. L. and B. T.; supervision, Y. L., A.M., and Christophe. L.; writing—original draft, Y. L., X. K., and O. S.; writing—review and editing, Y. L., A. M., B. T., Chrystèle. L., X. K., and V.C. All authors have read and agreed to the published version of the manuscript.

## FUNDING INFORMATION

This work was partly supported by the Grand Hôpital de l'Est Francilien.

## CONFLICT OF INTEREST STATEMENT

The authors declare no conflicts of interest.

## ETHICS STATEMENT

This study received a favorable advice from the “Comité de Protection des Personnes (CPP) Sud‐Ouest et Outre‐Mer”, n° IDRCB. 2018‐A01134‐51 (dossier 1–18‐42).

## CONSENT

A written consent was signed by the patient.

## Data Availability

The original contributions presented in this study are included in the article. Further inquiries can be directed to the corresponding author.
